# Activity-Dependent Shedding of the NMDA Receptor Glycine Binding Site by Matrix Metalloproteinase 3: A PUTATIVE Mechanism of Postsynaptic Plasticity

**DOI:** 10.1371/journal.pone.0002681

**Published:** 2008-07-16

**Authors:** Thorsten Pauly, Miriam Ratliff, Eweline Pietrowski, Rainer Neugebauer, Andrea Schlicksupp, Joachim Kirsch, Jochen Kuhse

**Affiliations:** 1 Department of Anatomy and Cellular Neurobiology, University of Ulm, Ulm, Germany; 2 Department of Anatomy and Cell Biology, University of Heidelberg, Heidelberg, Germany; University of Oldenburg, Germany

## Abstract

Functional and structural alterations of clustered postsynaptic ligand gated ion channels in neuronal cells are thought to contribute to synaptic plasticity and memory formation in the human brain. Here, we describe a novel molecular mechanism for structural alterations of NR1 subunits of the NMDA receptor. In cultured rat spinal cord neurons, chronic NMDA receptor stimulation induces disappearance of extracellular epitopes of NMDA receptor NR1 subunits, which was prevented by inhibiting matrix metalloproteinases (MMPs). Immunoblotting revealed the digestion of solubilized NR1 subunits by MMP-3 and identified a fragment of about 60 kDa as MMPs-activity-dependent cleavage product of the NR1 subunit in cultured neurons. The expression of MMP-3 in the spinal cord culture was shown by immunoblotting and immunofluorescence microscopy. Recombinant NR1 glycine binding protein was used to identify MMP-3 cleavage sites within the extracellular S1 and S2-domains. *N*-terminal sequencing and site-directed mutagenesis revealed S542 and L790 as two putative major MMP-3 cleavage sites of the NR1 subunit. In conclusion, our data indicate that MMPs, and in particular MMP-3, are involved in the activity dependent alteration of NMDA receptor structure at postsynaptic membrane specializations in the CNS.

## Introduction

The formation and the plasticity of synaptic structures of neurons are regulated by diverse sets of molecular mechanisms. Extracellular interactions of cell adhesion molecules and extracellular matrix proteins play a crucial role in initiating synapse- and receptor cluster formation by activating cellular signaling pathways [1]. Moreover, the activity of ligand gated ion channels contributes to the regulation of structural alterations of synaptic components. For example, it was shown that prolonged inhibition of *N*-methyl-D-aspartate (NMDA) receptor subtype of excitatory glutamate receptors can increase the number of NMDA receptor clusters by altered trafficking and synaptic targeting of receptor subunits in cultured neurons [2]–[4]. In general, these regulatory processes are thought to be dependent on receptor mediated ion fluxes, however, alteration of NMDA receptor numbers also occurs upon conformational changes of subunits induced by binding of the receptor coagonist glycine [5] to the extracellular S1S2-region of NMDA receptor NR1 subunits [6], [7].

The major structural determinants of targeting NMDA receptors and alpha-amino-3-hydroxy-5-methyl-4-isoxazolepropionic acid (AMPA) receptors to synapses are molecular signatures at intracellular carboxy-terminal sequence motifs [8]. For example, the two splice variants (NR1-C2 and NR1-C2′) of the carboxy-terminal region of the NMDA receptor NR1 subunits determine differentially the number and localization of NMDA receptor clusters in cultured neurons [3], [4], [9]. Interestingly, it was shown recently that two proteins (ABP and GRIP) which interact with the carboxy-terminus of AMPA receptors, bind to the membrane-type 5 matrix metalloproteinase (MT5-MMP), thereby directing MT5-MMP to growth cones and synaptic sites in neurons, where the proteolytic activity of this protein may be involved in remodelling of synapses by cleavage of cadherins and matrix proteins [10].

In the present study we used cultured spinal cord neurons to study alterations of postsynaptic NMDA receptor clusters under conditions of prolonged NMDA receptor activation. Using antibodies directed against extracellular or intracellular epitopes of the NR1 subunit, we found that chronic NMDA receptor activation leads to an exclusive loss of extracellular domains of NMDA receptor NR1 subunits. Furthermore, our data indicate that matrix metalloproteinase 3 is involved in proteolytic cleavage of the extracellular glycine binding site of the NR1 subunit.

## Results

### Long lasting stimulation of cultured spinal cord neurons with NMDA induces selective loss of NR1 extracellular epitopes

**Figure 1:**
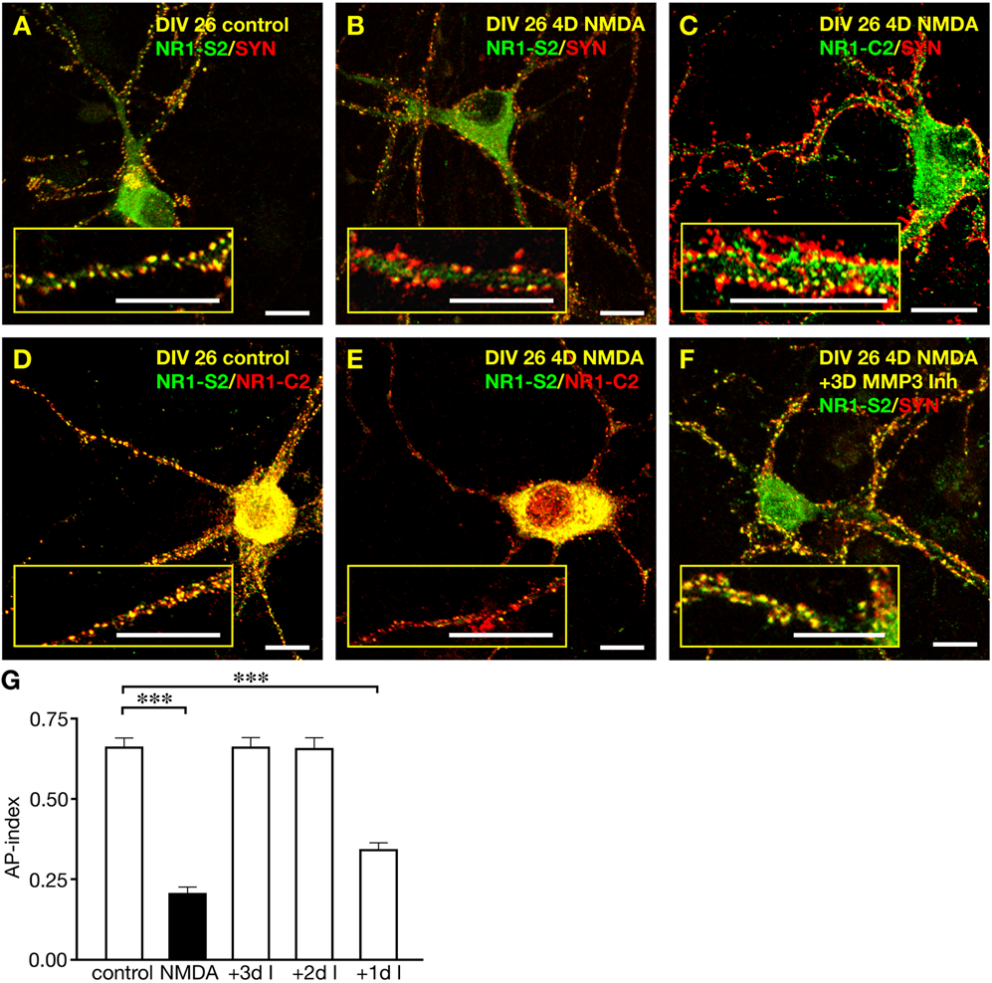
Chronic stimulation of spinal cord neurons with NMDA induces selective loss of extracellular epitopes due to MMP-3 activity. (A, B) At DIV26, cultured spinal neurons were fixed and stained with antibodies directed against NR1-S2 (green) and synaptophysin (red), revealing a reduction of postsynaptic NR1 puncta (yellow) upon NMDA treatment. Scalebar is 10 µm. (C) NMDA treatment did not reduce apposition of intracellular NR1-C2-epitopes (green) with synaptophysin (red), resulting in yellow signals. (D, E) Double-staining with NR1-S2 (green) and NR1-C2 (red) antibodies of control cells (D) and NMDA treated cells (E), show a selective loss of the extracellular NR1-S2 epitope upon receptor activation. (F) Immunofluorescence of DIV26 neurons, which have been treated with 10 µM NMDA in the presence of MMP-3 inhibitor (3 days), reveals NR1-S2 epitope detection (green) appositioned with synaptophysin (red). (G) Plot of apposition indices after chronic stimulation of DIV22 neurons with NMDA for 4 days. The reduction of apposition indices is prevented by MMP-3 inhibitor treatment for 3 (+3dI) or 2 days (+2dI), but not for 1 day (+1dI).

To study the alteration of receptor clusters upon NMDA stimulation we treated neurons cultured for 22 days in vitro, (DIV22) for four days with 10 µM NMDA. Postsynaptic NMDA receptors were analysed by immunofluorescence microscopy upon single and double detection experiments with antibodies specific for the presynaptic marker synaptophysin (detected with Cy3-conjucated second antibody, red fluorescence) and with antibodies directed against the extracellular S2-domain of the NR1 glycine binding site (Cy2-conjugated secondary antibody, green fluorescence). In another set of experiments we used anti-synaptophysin staining in combination with antibodies specific for the intracellular C2-domain of the NR1 subunit, one of two different splice variants (C2 and C2′) of the very carboxy-terminus and which is the major splice variant in cultured spinal cord neurons [4]. In mature neurons, synaptophysin labelling is known to occur almost exclusively at presynaptic boutons [13]. Therefore, the overlap of synaptophysin and NR1-immunoreactivities is assumed to indicate postsynaptic localization of NR1 subunits whereas extrasynaptic NR1 immunoreactivity appears independent of synaptophysin immunoreactivity. We calculated the ratio of yellow puncta and the total number of synaptophysin puncta for a dendritic length of 30 µm in 50 cells from three different cultures for each experimental condition by blinded analysis as apposition-index (AP-index) (Fig. 1A, B). When comparing the number of synaptophysin puncta in control and NMDA-treated cells, no changes in presynaptic bouton number were observed. However, the number of punctated, synaptically localized immunoreactivities detected with an antibody directed against the extracellular domains of the NR1 subunit (NR1-S2) was strongly decreased in NMDA-treated cells. Interestingly, the AP-index was reduced from 0.66±0.03 (mean±SEM; n = 50 cells) for control cultures to 0.20±0.03 (mean±SEM; n = 50 cells, p<0.001; two-tailed t-test) upon prolonged NMDA receptor activation (Fig. 1G). Unexpectedly, the analysis of cells which were stained with synaptophysin antibody and an antibody directed against the intracellular carboxy-terminus of the NR1 subunit (NR1-C2) revealed that the punctate C2-immunoreactivity was still abundant after NMDA treatment and in close apposition with synaptophysin (Fig. 1C). This observation suggested the loss of S2-epitope of the NR1 subunit while the carboxy-terminus was still localized in postsynaptic membranes. In addition, NR1-specific immunoreactivity which was not in apposition to synaptophysin was detected with both antibodies (NR1-S2; NR1-C2), presumably representing NR1 subunits within the dendritic compartment, however we focussed our analysis on the pool of NR1 subunits overlapping with synaptophysin immunoreactivity.

In order to directly compare the distribution of extracellular and intracellular-epitopes and to verify the specificity of the NR1-S2 and NR1-C2-antibodies, we performed double detection experiments with the two antibodies. These experiments revealed as expected an almost complete overlap of both immunoreactivities in control cultures (Fig. 1D). This observation is consistent with former studies showing that the NR1 splice variant containing the C2-cassette is the predominant version of the two alternative carboxy-terminal isoforms NR1-C2 and NR1-C2′ in this cell culture system under control conditions (see also [4]). Consistent with our previous results, the colocalization-index (ratio of yellow signals and total S2-specific puncta) decreased drastically from 0.91±0.03 (mean±SEM; n = 5) in control cultures to 0.23±0.02 (mean±SEM; n = 5, p<0.0001; two-tailed t-test) upon NMDA treatment (Fig. 1D, E). These data are in agreement with the assumption that prolonged NMDA receptor stimulation leads to a significant loss of extracellular NR1 epitopes.

To test whether receptor activation upon ligand binding could induce masking of the S2-epitopes by conformational changes we varied the duration of NMDA receptor stimulation. Interestingly, already after two days of NMDA treatment a selective loss of extracellular S2-epitopes was visible in double detection experiments using NR1-S2 with either NR1-C2 or synaptophysin-specific antibodies. The loss of S2-epitopes was not seen after one day of NMDA receptor stimulation (data not shown), indicating that the disappearance of S2-epitopes upon 2–4 days of NMDA treatment is unlikely to result from a masking of S2-epitopes due to agonist-induced conformational changes of receptor subunits, because these conformational changes of epitopes should occur with a short time of exposure to the ligand.

### MMP activity is required for the NMDA-induced loss of extracellular NR1-epitopes

The disappearance of extracellular NR1 epitopes while intracellular C2-epitopes were preserved suggested that the ectodomains might be cleaved off by proteolytic activity. Matrix metalloproteinases (MMPs) are known to be involved in ectodomain shedding of membrane proteins [14]. Therefore we treated the cultured neurons with NMDA as before and added different inhibitors of matrix metalloproteinases into the culture medium. The presence of MMP-8 or both MMP-2 and MMP-9-inhibitors during NMDA stimulation did not prevent the loss of extracellular NR1 epitopes (data not shown). However, applying the MMP-inhibitor N-isobutyl-N-(4-methoxyphenylsulfonyl)-glycylhydroxamic acid (NNGH, 1.3 µM) for the last 2 or 3 days of NMDA treatment prevented the loss of S2-epitopes. Under these conditions, the AP-index (ratio of yellow signals and synaptophysin signals) was 0.66±0.04 (mean±SEM; n = 50 cells) and 0.66±0.03 (mean±SEM; n = 50 cells), respectively (Fig. 1F, G), i.e. very similar to control conditions. These results suggested that blocking of MMPs other than MMP-2, MMP-8 or MMP-9 prevented the cleavage of ectodomains of the NR1 subunit. Interestingly, the presence of NNGH only at day 4 of the NMDA treatment was not sufficient to restore the postsynaptic localization of S2-epitopes (AP-index: 0.34±0.03, mean±SEM; n = 50 cells). These data indicate that inhibition of MMPs for only one day in culture may not be sufficient to detect newly synthesised NR1 subunits in the postsynaptic membrane in neurons that have lost NR1 S2-epitopes.

### PKC induced alteration of NR1 subunits is not dependent on MMP-activity

**Figure 2:**
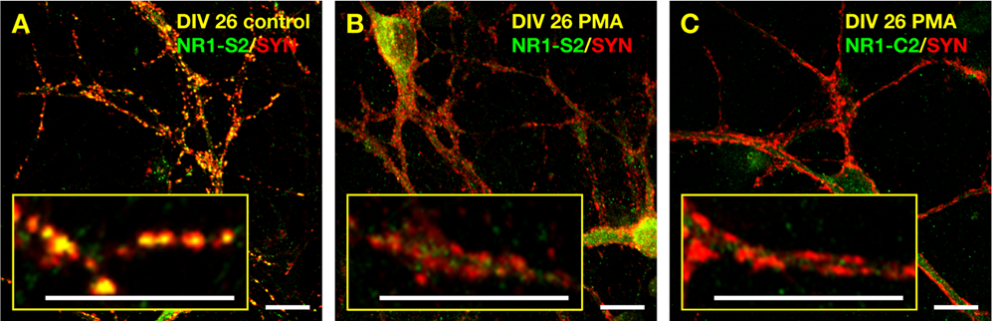
Inhibition of MMP-3 does not prevent NMDA receptor removal from synapses induced by PKC activation. (A) At DIV26, cultured spinal neurons were fixed and stained with an NR1-S2-specific antibody (green) and a synaptophysin-specific antibody (red), revealing a large number of postsynaptic NR1 puncta (yellow). Scalebar is 10 µm. (B) At DIV26, cultured spinal neurons were stimulated with 100 nM PMA for 30 min in medium supplemented with NNGH to inhibit MMP-3 activity, fixed and stained with an NR1-S2-specific antibody (green) and a synaptophysin-specific antibody (red), revealing a strong reduction of postsynaptic NR-S2 puncta (yellow) upon PKC activation. Scalebar is 10 µm. (C) At DIV26, cultured spinal neurons were stimulated with 100 nM PMA in medium supplemented with NNGH to inhibit MMP-3 activity, fixed and stained with an NR1-C2-specific antibody (green) and a synaptophysin-specific antibody (red), revealing also a strong reduction of postsynaptic NR1-C2-specific puncta (yellow) upon PKC activation. Scalebar is 10 µm.

To further analyse the observed NMDA induced loss of S2-epitopes we used other experimental conditions to reduce the number of NMDA receptor clusters. As described already for hippocampal neurons [15], PMA-mediated activation of protein kinase C (PKC) induced a fast reduction of the number of S2 and C2-specific clusters also in spinal neurons. The AP-index was reduced from 0.65±0.03 (mean±SEM; n = 5 cells) in control cultures to 0.19±0.02 (mean±SEM; n = 5 cells, p<0.0001; two-tailed t-test) and 0.10±0.02 (mean±SEM; n = 5 cells, p<0.0001; two-tailed t-test) for S2 and C2-immunoreactivities, respectively. Importantly, the disappearance of S2 and C2-epitopes was not prevented by the presence of MMP-inhibitor NNGH (Fig. 2B, C). These results are in agreement with the notion that the detected C2-immunoreactivity is specific for NR1 subunits and that the remaining C2-immunoreactivity after chronic NMDA stimulation is due to the presence of remnant C2-epitopes.

### MMP-3 digests solubilized NR1 subunits

**Figure 3:**
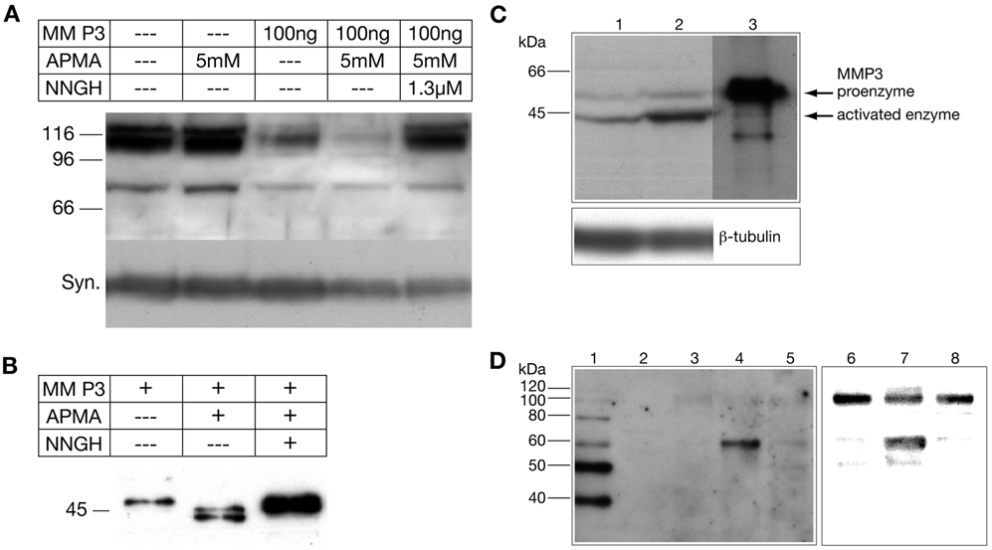
Analysis of MMP-3 and NR1 protein expression and digestion in cultured spinal cord neurons. (A) Upper panel: Solubilized proteins from rat brain membranes were incubated with MMP-3 proprotein and activated MMP-3 in the absence or presence of MMP-3 inhibitor NNGH and analysed by immunoblotting with a NR1-C2′-specific antibody. MMP-3 was activated by aminophenylmercuric acetate (APMA). Digestion of NR1 was prevented by the MMP-inhibitor NNGH. Lower panel: Immunoblot of the same probes with a synaptophysin-specific antibody (Syn.) revealing no strong degradation of this protein. (B) Immunoblot of S1S2M digestion assays (15h) with an MMP-3-specific antibody, demonstrating the presence of the active form of MMP-3 (45/44 kDa). (C) MMP-3 immunoblot of total protein extracts of cultured spinal cord neurons (DIV26) of control cells (lane 1) and cells treated with NMDA (4 days, 10 µM) (lane 2); for comparison, MMP-3 pro-protein (10 ng) was separated in parallel on the gel (lane 3). Lower panel: Immunoblot of the same gel as above probed with a β-tubulin antibody (loading control). (D) Detection of a 60 kDa proteolytic NR1 polypeptide on NR1 immunoblots (left panel, lanes 1–5; and right panel lanes 6–8). NR1 proteins were detected after separation of membrane proteins with SDS-PAGE and blotting using isolated membranes from spinal cord neurons cultured under control conditions (lane 3 and lane 6), from neurons treated with NMDA in the absence (lane 4 and lane 7), and presence of MMP-3 inhibitor NNGH (lane 5 and lane 8). The left panel was blotted with Na_2_-tetraborate buffer (Materials). Notice the clear detection of a protein band of about 60 kDa and the incomplete transfer of marker proteins larger than 50 kDalton (Magic Marker, Invitrogen, containing a protein domain detected by the secondary antibody). The right panel was blotted with methanol-containing buffer. Notice the clearer detection of an undigested NR1 protein band under these blotting conditions. Lanes 1 and 2 are loaded with protein molecular mass standards.

NNGH was originally described as inhibitor of MMP-3 [16], however, later other MMPs (for example MMP-10 or MMP-20) were shown to be inhibited by NNGH, although the affinity of NNGH for these MMPs are somewhat lower (43 fold and 6 fold lower, respectively) [17]–[18]. In order to analyse whether MMP-3 might be a candidate protease for to cleave the NMDA receptor NR1 subunit, we performed *in vitro* MMP-3 digestion assays with activated MMP-3 and solubilized membrane proteins from rat brain. As shown in Figure 3A, activated MMP-3 degraded solubilized NR1 subunits of about 110–120 kDa. Moreover, the presence of MMP-inhibitor NNGH in the digestion assays prevented proteolytic cleavage. An additional polypeptide of about 75 kDa is also recognized by the used antibody and is also degraded, however, whether this protein represents a NR1 fragment is not known. Immunoblotting of the *in vitro* assays with a synaptophysin-specific antibody revealed only a minor degradation of this protein indicating that the NR1 subunit of the NMDA receptor is a rather specific substrate of MMP-3. In control experiments we proved the activation of MMP-3 in the *in vitro* digestion assays by immunoblotting with an MMP-3-specific antibody, detecting the activated MMP-3 protein (44/45 kDa) (Fig. 3B).

To further corroborate the possible identification of NR1 as a MMP-3 substrate, we analysed MMP-3 and NR1-protein in cultured spinal cord neurons by immunoblotting and/or immunohistochemistry. Consistent with the hypothesis that MMP-3 is involved in the cleavage of NR1 ectodomains we detected the activated forms of MMP-3 (45 and ∼44 kDa) in addition to the MMP-3 proprotein (59 kDa) by immunoblotting experiments with total protein extracts from cultured spinal cord neurons. Densitometry revealed that the level of the activated MMP-3 was slightly increased upon NMDA treatment (1.54±0.13 fold, mean±SEM; n = 12) (Fig. 3C). In addition, we isolated membrane fractions from control cultures and from cultures treated with NMDA in the absence and in presence of the MMP-inhibitor NNGH and performed immunoblotting using anti-NR1-C2 together with anti-NR1-C2′-specific antibodies. In these experiments we detected a polypeptide of about 60 kDa in membrane preparations of NMDA treated spinal cord neurons but not from control cultures or upon using NNGH together with NMDA (Fig. 3C). The detection of the 60 kDa polypeptide was improved by shorter blotting times resulting in an incomplete transfer of “full-length” NR1-protein (Fig. 3D, left panel). Using other blotting conditions, the “full-length” NR1 protein in control cultures was clearly seen (Fig. 3D, right panel, lane 6). Moreover, we detected a reduced amount of undigested NR1 protein from cells, which were treated with NMDA compared to control conditions and those experiments in which NNGH-inhibitor was included (Fig. 3D, right panel). However, further cleavage reactions might occur, resulting in additional unstable proteolytic products which could not be detected under the experimental conditions which we used. Unfortunately, we could not detect N-terminal fragments of the NMDA receptor within the conditioned cell culture medium. This is possibly due to the low abundance or the instability of putative *N*-terminal-fragments.

**Figure 4:**
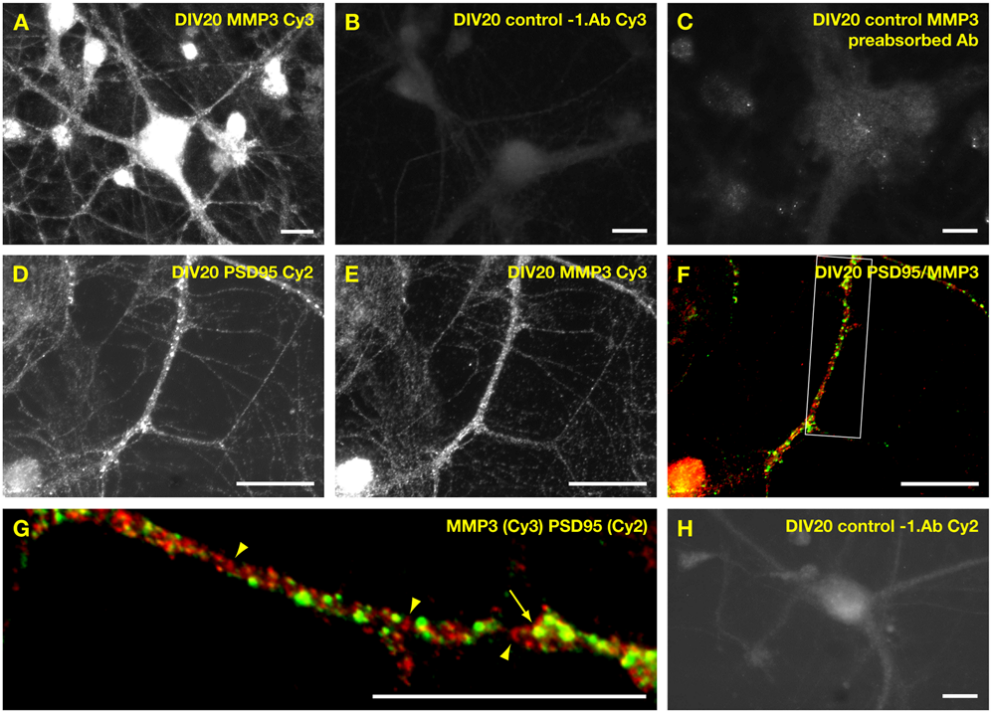
Expression of MMP-3 in cultured spinal cord neurons. (A) At DIV20, cultured spinal cord were fixed and stained with an MMP-3-specific antibody, revealing MMP-3 expression in soma, nucleus and neurites of spinal cord neurons. Scalebar is 15 µm. (B) Control experiments without first antibody using Cy3-conjugated anti-rabbit antibody. (C) Control experiments with anti-MMP-3 antibody which was preabsorbed with MMP-3 protein. Pictures (A–C) were taken under the same conditions (D) PSD-95-immunofluorescence of spinal cord neurons. (E) MMP-3-immunoreactivity of neurons shown in D. (F) Overlay of PSD-95 immunoreactivity (green) and MMP-3 immunoreactivity (red). Boxed region is shown as enlargement in G. (G) Enlargement of dendritic region shown in F. Arrowheads indicate accumulations of MMP-3 immunoreactivities within a dendrite. Arrow indicates overlapping PSD-95 and MMP-3-immunoreactivities. Scalebar is 15 µm (H) Control experiments using anti-mouse antibody conjugated with Cy2. Scalebar is 15 µm.

To analyse the cellular expression of MMP-3 we used an MMP-3-specific antibody for immunohistochemistry of cultured spinal cord neurons (Fig. 4). Interestingly, MMP-3 immunoreactivity was detected within the soma, the nucleus but also as discontinuous staining in dendrites of cultured spinal cord neurons (Fig. 4A). Control experiments without the MMP-3-specific antibody or with preabsorbed MMP-3 antibody revealed only low background staining (4B, C). The intensive nuclear staining detected in our experiments is in agreement with data from different recent publications showing convincingly that MMP-3 is localized in the nucleus in different tissues and cultured cell types [19], [20]. These studies have shown that MMP-3 contains functional nuclear localization signals and functions as transcription factor within the nucleus [20].

Double staining experiments using MMP-3-specific and PSD-95-specific antibodies showed that comparison of both immunoreactivities revealed only few overlapping puncta (Fig. 4, D–G), suggesting that most MMP-3 protein is localized at sites within dendrites distinct from PSD-95 containing synapses. In conclusion, our data are in agreement with the notion that cultured spinal cord neurons express MMP-3 which can be detected as proenzyme and as activated protein.

### MMP-3 cleaves the NR1 subunit within the glycine binding site *in vitro*

**Figure 5:**
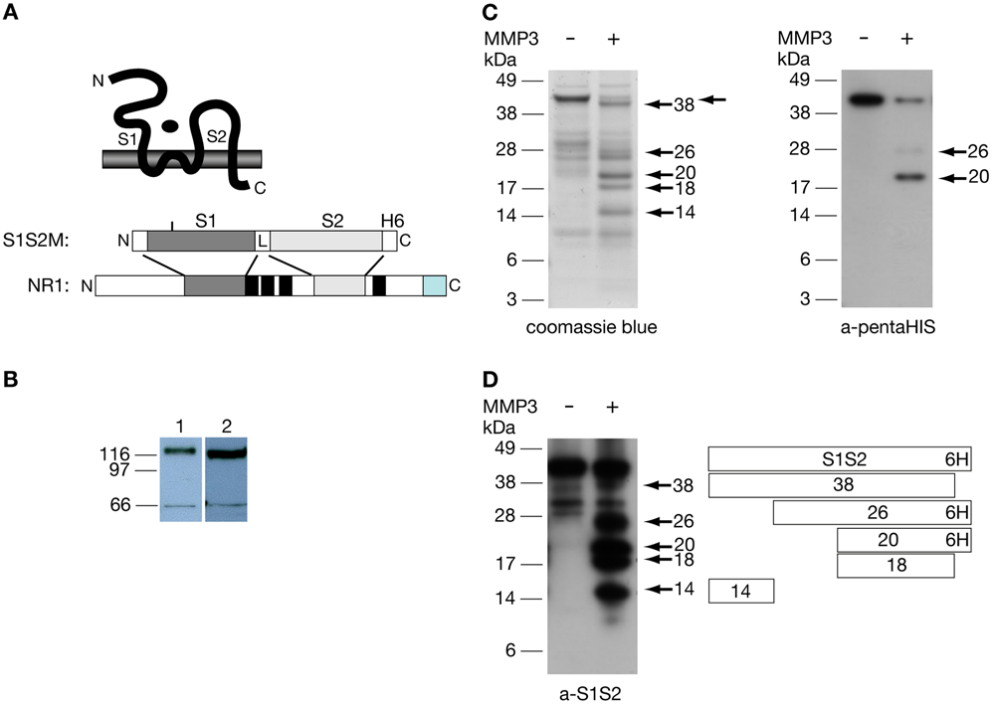
*In vitro* digestion of NR1 subunits with MMP-3. (A) Schematic representation of the subunit transmembrane topology, of the recombinant S1S2M protein and of the NR1 subunit. Black boxes indicate membrane domains and a grey box labels the specific carboxy-terminus, generated by alternative splicing. (B) Immunoblot with separated proteins from rat brain membranes with the novel S1S2-specific antibody (rabbit) (left site) and an NR1-S2-specific antibody (NMDAR, mouse, Chemicon). Both antibodies detect the NR1 subunit of about 120 kDa, in addition, a smaller fragment of about 66 kDa is seen. (C) Left side: Coomassie brillant blue staining of S1S2 protein fragments upon MMP-3 digestion. Protein fragments not seen in the control lane are indicated by arrowheads. The S1S2 protein is indicated by an arrow, and fragments are named according to their apparent molecular weights. Right side: Immunoblot with an anti-penta-HIS-antibody after *in vitro* digestion of S1S2M by active MMP-3. A major fragment of about 20 kDa and a minor fragment of about 26 kDa are indicated by arrows. (D) Immunoblot of S1S2M MMP-3 digestion experiments analysed by immunoblotting with the S1S2-specific antibody; right: a schematic view of putative proteolytic S1S2 fragments.

In order to further prove that NMDA receptor NR1 subunits might be cleaved by MMP-3, we performed *in vitro* MMP-3 digestion assays with activated MMP-3 and recombinant, soluble protein (S1S2M) that contained the extracellular glycine ligand binding site of the NR1 subunit (Fig. 5A) [12]. In order to detect proteolytic fragments of the recombinant protein we used an anti-S1S2 antibody and a penta-His antibody in immunoblot experiments. The specificity of the S1S2-antibody was established by immunoblotting experiments using isolated membranes from rat brain and affinity purified IgGs from immunized rabbit serum directed against S1S2 recombinant proteins (Fig. 5B). These experiments demonstrated that the novel rabbit antibody recognized the same major protein band of about 120 kDa which was also detected by an other characterized mouse antibody [21] specific for NMDA receptor NR1 subunits (Fig. 5B).

After incubation of recombinant S1S2M protein with active MMP-3, we detected cleavage products of ∼38 kDa, ∼26 kDa, ∼20 kDa, ∼18 kDa and ∼14 kDa after SDS-PAGE and Coomassie brillant blue staining (Fig. 5C). Immunoblotting with an anti-penta-His antibody (Fig. 5C right pannel), revealed a minor 26 kDa and a major 20 kDa protein fragment, which suggests that these polypeptides span from the S1 region to the His-tag at the carboxy-terminus of the S1S2 protein. Therefore the cleavage sites must be within the S1 domain. The additional fragments seen upon Coomassie staining (38 kDa, 18 kDa and 14 kDa) were identified as cleavage products of the S1S2 protein using an anti-S1S2 antibody for immunoblotting (Fig. 5D). By MALDI-TOF analysis, the 14 kDa and 18 kDa-fragments could be attributed to the S1 and S2-domains of the S1S2 polypeptide, respectively, because peptides obtained from these protein bands upon trypsin digestion corresponded to the respective sequences of the S1S2 protein (data not shown). In order to determine the cleavage sites within the S1S2 protein *N*-terminal sequencing of the 26 kDa, 20 kDa and 14 kDa fragments was performed. These experiments revealed that the 14 kDa fragment exactly matches the *N*-terminus of the expressed recombinant protein whereas the analysis of the major cleavage product of 20 kDa identified a serine within the S1 domain corresponding to serine 542 of the NR1 subunit as amino-terminal amino acid of the 20 kDa fragment and thus position S542 as one major MMP3 cleavage site within the NR1 subunit. However, different attempts of *N*-terminal sequencing of the 26 kDa protein band did not result in an unambiguous amino acid sequence.

**Figure 6:**
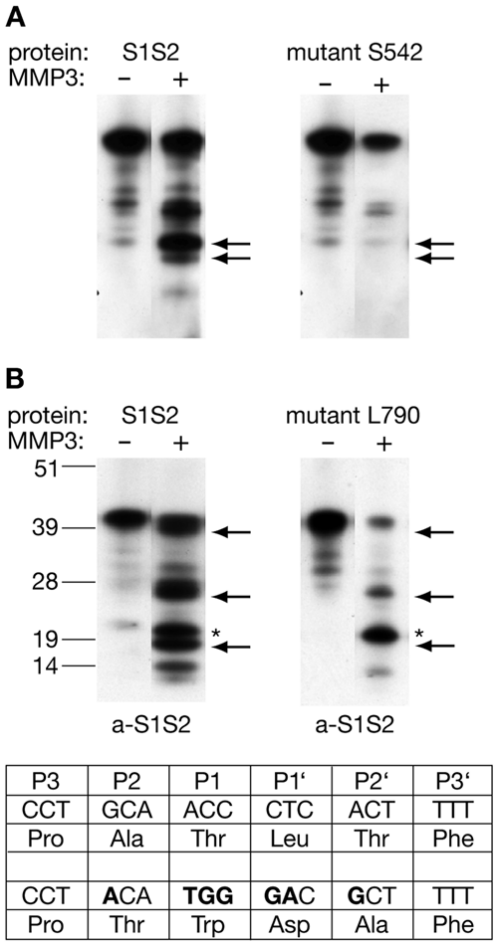
Analysis of MMP-3 cleavage sites by site-directed mutagenesis. (A) Immunoblot of S1S2 and mutant S1S2Ser542 protein fragments. S1S2 and mutant S1S2Ser542 proteins were purified, incubated with active MMP-3 and separated by SDS-PAGE. Immunoblotting with anti-S1S2 antibody revealed that the mutations abolished both the appearence of the 20 kDa and 18 kDa fragments (indicated by arrows), suggesting that both fragments are generated from the same *N*-terminal MMP-3 cleavage site. (B) Immunoblot of separated protein fragments generated upon MMP-3 digestion of S1S2- and mutant S1S2Leu790 proteins. Note the presence of three protein double bands of 40/38 kDa, 26/24 kDa and 20/18 kDa in the S1S2 protein digestion pattern (left), which are absent in the digestion pattern with mutant S1S2Leu790 protein (indicated by arrows). Note that the cleavage site generating the 20 kDa protein fragment (indicated by star) is not effected by this mutation, lower panel: mutated MMP-3 cleavage site of the S1S2Leu790 protein.

To further corroborate the identified site within the S1-region as the MMP-3 cleavage site, we altered the encoded amino acid sequence by site-directed mutagenesis of the expression construct. With this approach we generated a fusion protein in which S542 (S178 of the S1S2 construct) was replaced by aspartate (mutant S1S2Ser542). In addition, three amino acids arround serine 542 were replaced by different amino acid residues (see methods) because these P3-P1 positions are known to contribute to the specificity of MMP-3 cleavage [22]. The mutant S1S2Ser542 protein was expressed in *E.coli*, purified and renaturated as described [12] and MMP-3 *in vitro* digestion experiments were performed. As shown in Figure 6A the analysis of cleavage products by immunoblotting with anti-S1S2 antibody showed that the generation of the 20 kDa and 18 kDa-fragments by MMP-3 activity was completely abolished, suggesting that amino acids spanning from P539 at the P3 position to Q541 at position P1 and S542 at position P1′ of the NR1 subunit may by important for sequence-specific cleavage by MMP-3.

The existence of a protein fragment of about 38 kDa seen upon Coomassie staining of separated MMP-3 cleavage products, which was not recognized by the anti-penta-His antibody, indicated that one MMP-3 cleavage site should be localized near the very carboxy-terminus of the S1S2 protein. Because the resulting short *C*-terminal cleavage product was too small to be isolated by gel-electrophoresis and to be analysed by *N*-terminal sequencing we compared the corresponding region with regard to sequence similarities with known MMP-3 cleavage sites of other proteins. L790 of the NR1 subunit was selected as a candidate cleavage site because leucine is the most conserved amino acid residue within known MMP-3 cleavage sites. In addition, assuming L790 to be positioned at the P1′ site of a putative cleavage site, a proline and an alanine would be positioned at P3 and P2 positions, respectively, which again is a favoured configuration within known MMP-3 cleavage motifs [22]. Therefore, we altered this sequence motif by site-directed mutagenesis of the corresponding expression construct, purified and renaturated the resulting mutant S1S2Leu790 protein and performed *in vitro* MMP-3 digestion experiments. As seen in Figure 6B, the analysis of cleavage products by immunoblotting experiments with anti-S1S2 -antibody showed that the 38 kDa and 18 kDa-protein fragments could not be detected, whereas the detection of the 20 kDa polypeptide was not altered. In addition, a band detected within the control reaction running faster than the 26 kDa band was not seen at the respective position with the mutant S1S2Leu790 protein. These results are consistent with the assumption that the mutated sequence motif at the carboxy-terminus of the S1S2 protein constitutes a MMP-3 cleavage site. Thus, in the mature NR1 subunit a second MMP-3 cleavage site should be localized within the sequence P787-L790, most likely between T789 and L790.

## Discussion

In the present study we demonstrate that (*i)* prolonged NMDA receptor stimulation resulted in the loss of extracellular epitopes of the NMDA receptor NR1 subunit, (*ii)* blocking of MMPs activity inhibited this process, *(iii)* MMP-3 specifically cleaved the NR1 protein *in vitro*, in particular the extracellular glycine binding domains, *(iv)* S542 and L790 of the NR1 subunit are important determinants of two MMP-3 cleavage sites within this NMDA receptor subunit. These data suggest that the matrix metalloproteinase MMP-3 is involved in the shedding of ectodomains of the NMDA receptor NR1 subunit. We identified at least three MMP-3 cleavage sites within the S1 and S2-domains. One MMP-3 cleavage site could be determined by *N*-terminal sequencing at position S178 of the S1S2 protein, corresponding to S542 of the NR1 subunit, a position exactly *N*-terminal to the first NR1 transmembrane region. A second MMP-3 cleavage site was identified in the carboxy-terminal region of the S1S2 protein, corresponding to a position directly *N*-terminal to the fourth transmembrane region of the NR1 subunit, consistent with the observation that S2-specific epitopes are abolished upon prolonged NMDA receptor activation. The proteolytic digestion of the NR1 subunit is likely to remove the cleaved domains of the glycine binding pocket of the NMDA receptor and thereby renders the receptor non-functional [23].

To our knowledge this is the first study showing that processing of the extracellular domains of NMDA receptors by matrix proteins could be involved in alteration of the NMDA receptor structure. The identification of MMP-3 as a potential regulatory component of synapse structure and function is consistent with the increasing evidence that components of the extracellular matrix play important roles in the regulation of neuronal connectivities. For example, it is already well established that the perisynaptic net of extracellular matrix components is important for determining boundaries of axonal fields in the dentate gyrus [24]. Interestingly, inhibition of MMP-3 and MMP-9 was shown recently to effect spatial learning and synaptic plasticity in rats [25]. For the neuro-muscular junction it was shown that MMP-3 specifically removes agrin from the synaptic basal lamina [26], thereby regulating the structure of the neuromuscular junction [27]. In the CNS, a specific agrin isoform is expressed [28], which might be involved in glutamatergic signaling [29]. Interestingly, also the neuronal isoform of agrin is cleaved by MMP-3 in ischemic neuronal tissue of rats after transient middle cerebral artery occlusion [30]. As it was shown, that MMP-3 activation in neuronal tissue during trauma-induced synaptogenesis is dependent on NMDA receptor activity [31], it is conceivable that MMP-3 activation *in vivo* occurs under conditions of extensive glutamate receptor activation, similar to the conditions of extended stimulation of NMDA receptors in our *in vitro* experiments. Indeed, more recently it was shown that activated MMP-3 is released from apoptotic neuronal cells. Importantly, it was demonstrated that the released and proteolytically activated MMP-3 acts as signaling molecule to activate microglia, which in turn might play a role in exacerbating degenerative human brain disorders, such as Parkinson's disease [32]. One other study has identified the membrane-bound type 5 matrix metalloproteinase to interact with postsynaptic density proteins and to be enriched at glutamatergic synapses, where it may be involved in cleavage of the cell adhesion molecule cadherin, thereby possibly contributing to synaptogenesis and activity-dependent synaptic remodelling [10].

Classical substrates of MMP-3 include extracellular macromolecules like collagen IV, elastin and fibronectin [33]. Later studies have identified additional substrates such as osteopontin [34] and SPARC (Secreted Protein, Acidic and Rich in Cysteine), a glycoprotein that modulates cell proliferation, adhesion, migration, and extracellular matrix production [35]. Interestingly, the cleavage of extracellular domains of transmembrane proteins like the CD95/APO-1/Fas ligand could also be demonstrated [14]. The latter finding indicates that MMP-3-dependent shedding of this signaling molecule is involved in regulating apoptotic cell death.

Similarly, MMP-3 cleavage of the NMDA receptor NR1 subunit might be related to neuronal cell death. We propose a model in which MMP-3 may be activated under conditions of strong NMDA receptor stimulation *in vivo* and MMP-3-mediated NR1 subunit inactivation by ectodomain shedding may antagonize the over-stimulation of the NMDA receptors in order to prevent neuronal cell death. In addition, MMP-3 could be involved in synaptic remodelling of excitatory synapses by cleavage of extracellular matrix proteins. This process might be regulated by NMDA receptor activity, as already indicated by different *in vivo* studies [30], [31]. Future research will reveal by which cellular mechanisms the activity of MMP-3 is regulated and whether MMP-3 may be released at specific synaptic sites in order to allow local modifications of the perisynaptic network and of synaptic proteins like neuronal agrin and NMDA receptors, thereby contributing to increased synaptic plasticity.

## Methods

### Tissue preparation

Spinal cord neurons were prepared from embryonic stage 14 (E14) rats (Wistar and Sprague-Dawley (SD) strains; Charles River, Sulzbach Germany) as described earlier [11]. Animal care was in accordance with the European Communities Council Directive (86/609/EEC) to minimize pain or discomfort. Spinal cord neurons were plated on glass coverslips (14 mm, density: 5×10^4^ cells/well). Cells were maintained in culture medium (Neurobasal medium with B27-supplement) (Gibco, Karlsruhe, Germany) until the indicated time points (days *in vitro*; DIV).

### Treatment of cells

Treatment of cells was performed for four days (DIV22–DIV26) by daily addition of NMDA (final concentration 10 µM) into the medium. The following MMP-inhibitors were used in a final concentrations of ∼10 fold of their individual Ki-values: MMP2/MMP9 inhibitor ((2R)-2-((4-biphenylylsulfonyl)amino)-3-phenylpropionic acid) 3.25 µM, MMP8 inhibitor ((3R)-(+)-(2-(4-methoxybenzenesulfonyl)-1,2,3,4-tetrahydro-isoquinoline-3-hydroxamate) 50 nM and MMP-3 inhibitor (n-isobutyl-N-(4-methoxyphenylsulfonyl-glycylhydroxamic acid (NNGH)) 1.3 µM. All inhibitors were purchased from Calbiochem.

### Immunostaining

For immunostaining with NMDA receptor NR1 subunit-specific antibodies cells were fixed and permeabilized on coverslips using acetone (−20°C) for 2 minutes. Unspecific binding was blocked by incubation in 1% (w/v) bovine serum albumine (BSA) in phosphate buffered saline (PBS) pH 7.9 at RT for 60 minutes. Incubation with the primary antibody was performed for 60–120 minutes at RT in a humidified chamber. For immunostaining with a rabbit anti-MMP-3 antibody (Cat. No. M 4802, Sigma) spinal cord neurons were fixed with methanol (−20°C) for 10 minutes. Unspecific binding was blocked with 1% (w/v) BSA and 5% horse serum (v/v), 0.15% (v/v) Triton in PBS for 1h at RT. Anti-NMDAR antibody (NR1-S2) (mouse, Cat. No. MAB363, 1∶100) and anti-NR1-C2 antibody (rabbit, Cat. No. AB5050P, 1∶50) were purchased from Chemicon (Temecula, USA). Anti-PSD95 antibody (mouse, Cat. No. 05. 494, 1∶500) was purchased from Upstate. Anti-synaptophysin antibodies (rabbit, Cat. No. 101 002, Synaptic Systems, Göttingen, Germany), (mouse, SVP-38, Cat. No. S 5768, Sigma, Taufkirchen, Germany) were used at final dilutions of 1∶200. The antibodies were diluted as indicated in 1x PBS (pH 7.9) with 1% (w/v) BSA prior to use. Coverslips were washed three times (5 minutes each) in PBS and incubated with fluorochrome (Alexa488, Cy2, Cy3)-conjugated secondary antibodies for 30 minutes in a dark humidified chamber. After three additional washes in PBS (pH 7.9, 5 minutes each) in the dark, coverslips were mounted in Mowiol and stored at 4°C in the dark until microscopical evaluation. Secondary antibodies were from Molecular Probes (Eugene, USA). All secondary antibodies were used at final dilutions of 1∶800 in PBS with 1% (w/v) BSA. Staining experiments without or with “cross-species”-primary antibodies were performed to ensure the specificity of the secondary antibodies. Preabsorbtion of anti-MMP-3 antibody was done in 1∶10 molar excess of MMP-3 protein for 30 minutes at RT.

### Microscopical evaluation

Images of NMDA receptor analysis were recorded using a Leica TCS-NT confocal laser scanning microscope equipped with a 100× oil-immersion lens (Leica) with a numerical aperture of 1.35. An argon-krypton laser was used for excitation with 488 nm and 568 nm lines and emission-windows were set to 500 nm–535 nm and 585 nm–620 nm, for the detection of green and red fluorescence, respectively. Laser intensities and photo-multiplier settings were used in a way that no cross-excitation was detected. Images were recorded in a sequential channel mode and were acquired as multiple Z-slices using an image size of 1024×1024 pixels. Individual sections were scanned twice and averaged to optimize signal-to noise ratios. Extended focus projections were calculated from individual sections and used to generate overlays of both fluorescence channels. The apposition index was calculated as ratio of yellow puncta and total synaptophysin specific (red and yellow) puncta or as colocalization index of C2 and S2-imunoreactivities as ratio of yellow puncta and total S2-specific puncta. MMP-3 detection experiments were analysed using a Zeiss Axiovert 200M motorized microscope (Zeiss, Jena, Germany) attached to a SPOT cooled CCD camera (Visitron Systems, Puchheim, Germany). All images were collected using 63× or 100× Plan-Neofluar oil objectives.

### Statistical analysis

Statistical analysis was performed using the GraphPad-Prism IV software as indicated in the Results section. Values of p<0.05 were considered as significant.

### Isolation of membranes from cultured neurons and solubilization of membrane proteins from rat brain

For preparation of membranes from cultured spinal cord neurons, cells were washed three times with PBS-N (Gibco) and subsequently scraped off from 6 well-plattes using a disposable cell scraper (Greiner) with 0.5 ml/well potassium-phosphate buffer (25 mM), pH 7.0. containing a mixture of proteinase-inhibitors (protease inhibitor complex complete, Boehringer, Germany; phosphoramidon (330 µg/ml) and GM6001, 2.6 µM). Cells were homogenized using a Potter-Elvehjem glass homogenizer at 4°C. The homogenate was centrifuged first for 15 min (4°C, 1.000× g) and the supernatant was again centrifuged for 45 min at 4°C at 120.000× g. The membrane pellet was resuspended in 2× Laemmli SDS-PAGE loading buffer. For total protein extracts, cells were lysed directly on the cell culture dish with 4× Laemmli SDS-PAGE loading buffer.

For solubilization of membrane proteins rat brains (1 g, adult rat, Wistar) were homogenized using a Potter-Elvehjem glass homogenizer at 4°C in 50 mM Tris-HCl, pH 9.0. The homogenate was centrifuged first for 15 min (4°C, 1.000× g) and the supernatant was then centrifuged for 30 min at 4°C at 30.000× g. The membrane pellet was resuspended in 50 mM Tris pH 9.0 supplemented with 1% (w/v) deoxycholate. Solubilization of membrane proteins was performed for 1 hr at 37°C with permanent shaking. Non-solubilized material was cleared by ultracentrifugation at 100.000× g for 1 hr at 4°C.

### Site-directed mutagenesis

The plasmid expression construct S1S2M [12] was used as a template for site-directed mutagenesis. Note that the construct includes threonine 543 of the S1 domain, numbering counts the amino acid position of the putative mature protein assuming that the sequence Arg-Ala-Ala represents the first three N-terminal amino acids upon cleavage of the signal peptide of the NR1 subunit. Site-directed mutagenesis was performed using the “Quick Change XL-Site-Directed Mutagenesis Kit” (Stratagene, USA). The sequences of all constructs were verified by DNA sequencing. For generation of mutant S1S2Ser542 the following sense oligonucleotide together with the respective complementary anti-sense oligonucleotide was used: 5′-gagcacactggactcatttatgcagcgtcattgggacaccgagggtggggtcaatgccgaag-3′. The encoded sequence of mutant construct contained the amino acid sequence Arg^539^-His-Trp-Asp^542^ replacing the sequence Pro^539^-Phe-Gln-Ser^542^ of the “wild type” S1S2M protein sequence. For the construction of mutant S1S2Leu790 we used the following sense oligonucleotide 5′-gactcccgcagcaatgctcctacatgggacgcttttgagaaccatcaccaccatc-3′ together with the complementary anti-sense oligonucleotide, resulting in an alteration of four amino acid positions surrounding L790: Ala^788^-Thr-Leu-Thr^791^ were replaced by the sequence Thr^788^-Trp-Asp-Ala^791^

### In vitro MMP-3 digestion

Solubilized membrane proteins were treated with 4 M urea, 50 mM Tris pH 7.0 for 0.5 hr at 37°C, dialyzed at 4°C against 2.500 volumes of MMP-3 digestion buffer (50 mM Tris-HCl pH 7.5, 200 mM NaCl, 10 mM CaCl_2,_ 20 mM ZnCl_2_) for 2.5 h. MMP-3 (100 ng, Chemicon, Temecula, USA) was activated with 1.5–5 mM 4-aminophenyl-mercuricacetat (APMA) for 2 hr at 37°C and subsequently incubated with 50 µg of resuspended membrane proteins in a final volume of 20 µl for 15 hr at 37°C. Reactions were stopped by adding SDS-PAGE loading buffer and reaction products were analysed by SDS-PAGE and immunoblotting. Recombinant S1S2M protein was expressed in *E. coli*, isolated and renaturated as described [12] dialysed against 2.500 volumes of MMP-3 digestion buffer and denaturated protein was separated by ultracentrifugation at 100.000× g for 1 h at 4° C. Soluble protein (750 ng) was incubated with 75 ng of APMA-activated MMP-3 (2 h, 37°C) or with 75 ng MMP-3 catalytic domain (Calbiochem) for 4–15 h at 37° C.

### N-terminal sequencing and MALDI-TOF mass spectrometry

N-terminal sequencing of S1S2-fragments was performed by a commercial supplier (TopLab, Martinsried) using a proteinsequencer Procise 492 (Applied Biosystems). MALDI-TOF mass spectrometry analysis was performed by the core facility for Mass Spectrometry and Proteomics (ZMBH, University of Heidelberg).

### Generation of an anti-S1S2 antibody

S1S2 protein was expressed in *E. coli*, purified and renaturated as described [12] and used for immunisation of rabbits (BioScience, Göttingen, Germany). The IgG fraction was purified by salt precipitation of sera obtained from final bleedings after two booster antigen injections and subsequently by immunopurification using S1S2 protein bound to Sulfo-link^TM^ matrix (Pierce, Rockford, USA). Monospecific antibodies were stored in 50% (w/v) glycerol stocks at −20° C and used for immunoblotting at a dilution of 1∶200.000.

### Immunoblot analysis

Products of *in vitro* MMP-3 digestion, protein extracts from cultured spinal cord neurons and proteins from isolated membranes from cultured spinal cord neurons were separated by 10% polyacrylamide (w/v) SDS-gels or 4%–12% (w/v) polyacrylamide gradient NuPAGE gels (Invitrogen, Carlsbad, CA, USA) and proteins were blotted on polyvinylidene difluoride (PVDF) membranes (Millipore, Billerica) according to the manufacturers' instructions using either Na_2_-tetraborat buffer (10 mM) or SDS-PAGE -running buffer supplemented with 20% methanol. Membranes were probed with anti-NR1-C2 antibody (rabbit, 1∶2000, AB5050P, Chemicon, Temecula, USA), anti-S1S2 antibody (rabbit, 1∶40.000), anti-Penta-His-antibody (mouse, 1∶10.000, Qiagen), anti-synaptophysin antibody (rabbit, 1∶10.000, Cat. No. 101 002, Synaptic Systems, Göttingen, Germany) and mouse anti-β tubulin antibody (1∶50.000, Cat. No. T 0198, Sigma, Taufkirchen, Germany). MMP-3 proteins were detected with mouse anti-MMP-3 antibody (1∶4.000, MAB1339, Chemicon, Temecula, USA). HRP-linked secondary antibodies were visualized using ECL-Plus (Amersham Pharmacia, Freiburg, Germany). After exposure films were developed and signals were scanned and analysed using Kodak Digital science 1D software.
